# Characterisation of QTL-linked and genome-wide restriction site-associated DNA (RAD) markers in farmed Atlantic salmon

**DOI:** 10.1186/1471-2164-13-244

**Published:** 2012-06-15

**Authors:** Ross D Houston, John W Davey, Stephen C Bishop, Natalie R Lowe, Jose C Mota-Velasco, Alastair Hamilton, Derrick R Guy, Alan E Tinch, Marian L Thomson, Mark L Blaxter, Karim Gharbi, James E Bron, John B Taggart

**Affiliations:** 1 The Roslin Institute and Royal (Dick) School of Veterinary Studies, University of Edinburgh, Midlothian, EH25 9RG, UK; 2 Institute of Evolutionary Biology, Ashworth Laboratories, King’s Buildings, University of Edinburgh, Edinburgh, EH9 3JT, UK; 3 Landcatch Natural Selection Ltd., Alloa, Clackmannanshire, FK10 3LP, UK; 4 The GenePool Genomics Facility, Ashworth Laboratories, King’s Buildings, University of Edinburgh, Edinburgh, EH9 3JT, UK; 5 Institute of Aquaculture, University of Stirling, Stirling, FK9 4LA, UK

**Keywords:** Atlantic salmon, RAD sequencing, Aquaculture, Infectious pancreatic necrosis, Recombination, Single nucleotide polymorphism, Paralogous sequence variant

## Abstract

**Background:**

Restriction site-associated DNA sequencing (RAD-Seq) is a genome complexity reduction technique that facilitates large-scale marker discovery and genotyping by sequencing. Recent applications of RAD-Seq have included linkage and QTL mapping with a particular focus on non-model species. In the current study, we have applied RAD-Seq to two Atlantic salmon families from a commercial breeding program. The offspring from these families were classified into resistant or susceptible based on survival/mortality in an Infectious Pancreatic Necrosis (IPN) challenge experiment, and putative homozygous resistant or susceptible genotype at a major IPN-resistance QTL. From each family, the genomic DNA of the two heterozygous parents and seven offspring of each IPN phenotype and genotype was digested with the SbfI enzyme and sequenced in multiplexed pools.

**Results:**

Sequence was obtained from approximately 70,000 RAD loci in both families and a filtered set of 6,712 segregating SNPs were identified. Analyses of genome-wide RAD marker segregation patterns in the two families suggested SNP discovery on all 29 Atlantic salmon chromosome pairs, and highlighted the dearth of male recombination. The use of pedigreed samples allowed us to distinguish segregating SNPs from putative paralogous sequence variants resulting from the relatively recent genome duplication of salmonid species. Of the segregating SNPs, 50 were linked to the QTL. A subset of these QTL-linked SNPs were converted to a high-throughput assay and genotyped across large commercial populations of IPNV-challenged salmon fry. Several SNPs showed highly significant linkage and association with resistance to IPN, and population linkage-disequilibrium-based SNP tests for resistance were identified.

**Conclusions:**

We used RAD-Seq to successfully identify and characterise high-density genetic markers in pedigreed aquaculture Atlantic salmon. These results underline the effectiveness of RAD-Seq as a tool for rapid and efficient generation of QTL-targeted and genome-wide marker data in a large complex genome, and its possible utility in farmed animal selection programs.

## Background

Atlantic salmon (*Salmo salar* L.) is a species of economic importance to both wild fisheries and aquaculture production. The worldwide production of Atlantic salmon through aquaculture has increased rapidly over recent years, and is now approximately 1.5million tonnes per annum [1]. Large-scale salmon breeding programs were first established in the 1970s [2], and the typical four-year generation interval means that selected lines of farmed salmon remain just a few generations from ancestral wild fish [3]. Family-based selection has resulted in rapid improvement in economically important traits, including growth, age at maturation and resistance to pathogens [4] . Genetic markers have been critical for this process, and are often utilised for family assignment and mapping loci of economic importance. Microsatellite and SNP resources are available for Atlantic salmon [5-8], and the ongoing genome sequencing project is anticipated to result in further genetic marker discovery [9]. However, the genomics resources available still lag behind terrestrial livestock species, which hinders the application of genomic selection [10,11] and fine-scale analysis of the genomic regulation of scientifically and economically important traits. Furthermore, due to the diverse ancestral origins of different breeding programmes, marker assays developed from a single reference genome or a limited set of genomes may not be fully informative in a population or family of interest.

Advances in high-throughput short-read sequencing technology have facilitated rapid and cost-efficient generation of gigabases of data by individual laboratories. This has led to new approaches for simultaneous discovery and genotyping of dense genetic markers on a scale that represents a step change from the state of the art prior to this technology [12]. One such approach is Restriction-site Associated DNA (RAD) sequencing (RAD-Seq), which is a genome complexity reduction technique that sequences at depth flanking regions of restriction enzyme cleavage sites [13]. This enables reliable base calling and SNP identification. RAD-Seq is typically applied to multiplexed samples, where DNA fragments are ligated to a sample-specific barcode sequence for pooled sequencing, with subsequent *in silico* assignment of reads to samples enabling individual and population-level genotyping [13,14]. RAD-Seq data can be readily analyzed without a reference genome, which makes the technique particularly applicable to non-model organisms, including Atlantic salmon. It can be applied *in lieu* of or in addition to other SNP assay technologies, because RAD-Seq can generate a large-scale population-specific marker set with individual genotypes.

One of the main foci of QTL mapping studies in Atlantic salmon to date has been the genetic regulation of host resistance to disease [15-20]. The most striking example is the discovery of a locus explaining almost all of the genetic variation in resistance to Infectious Pancreatic Necrosis [15,17]. IPN is a viral disease, the causal agent of which is a highly contagious birnavirus that can cause high levels of mortality at both the early freshwater and sea water stages of the salmon lifecycle [21]. An individual's possession of one or two favourable copies of the resistance allele markedly reduces the likelihood of mortality, the magnitude of which is dependent on the severity of the overall epidemic. However, studies by Scottish and Norwegian research groups have independently verified the large difference in mortality between alternate QTL homozygotes in both controlled challenge and ‘field’ seawater exposures, with estimates of mortality proportion ranging from 0.36 to 1 [15,17,22,23]. The resistance allele has been selected for in breeding programs using marker-assisted selection [17,22], but the causal gene and mutation remain elusive. The position of the QTL has been narrowed to a 3 cM confidence interval on linkage group 21 [17]. However, locating and investigating the underlying gene(s) requires a higher density of genetic markers within the QTL region than is currently available.

The salmon genome, in common with other salmonid species, has undergone a duplication event 25–100 MYA, and it demonstrates residual tetrasomic inheritance. This is evidenced by extensive gene duplication and homeology across much of the genome and quadrivalent formation at meiosis in males [24,25]. A related complication in salmonid genomics is the high number of paralogous sequence variants (PSVs) which can appear as putative SNPs in sequence alignment analysis, and need to be distinguished from true segregating SNPs. For example, Hohenlohe *et al.* used excess heterozygosity to exclude PSVs from RAD-derived SNP panels generated for both rainbow trout (*Oncorhynchus mykiss*) and cutthroat trout (O. *clarkii*) [26], while Sanchez *et al.* utilised double-haploid rainbow trout to filter out PSVs discovered using reduced representation sequencing [27]. Additionally, there is extreme heterochiasmy in salmon, with very little recombination over large sections of the genome in males, while females demonstrate more typical patterns of crossover and recombination. This is evident in the linkage maps that have been created for Atlantic salmon to date, where ratios of female:male recombination rates have been estimated at between 1.4:1 and 16.9:1 [5-8,28]. The most recent Atlantic salmon linkage map highlights a large difference in the genomic distribution of male and female recombination, with male recombination more common near putative telomeres [8]. A recent study using RAD-Seq also demonstrated a general lack of male recombination in rainbow trout by the clustering of the majority of markers proximal to putative centromeric positions in the linkage map [29].

In the current study, our aim was to discover, verify and genotype a large number of polymorphic markers in the Atlantic salmon genome, with a particular focus on markers linked to the IPN resistance QTL. By applying RAD-seq to parent and offspring samples, we aimed to verify marker segregation and help identify and distinguish between SNPs and PSVs. We also utilised the lack of salmon male recombination to analyse genome-wide patterns of RAD marker segregation from sires to offspring to identify putative linkage groups. To identify trait and QTL-linked markers, we used heterozygous parents and their offspring of known IPN resistance phenotype and QTL genotype (putative homozygous resistant or homozygous susceptible) to identify candidate resistance and susceptibility alleles though linkage. A subset of trait and QTL-linked SNP markers were then verified using a high-throughput assay applied to larger populations of IPNV-challenged Atlantic salmon.

## Results

### RAD-seq data processing

Two families were identified from an earlier QTL mapping experiment as having both sire and dam heterozygous for the IPN resistance QTL [22]. Using microsatellite markers from Atlantic salmon linkage group 21 (corresponding to chromosome 26 [30]), seven offspring from each family were classified as homozygous for the resistance allele and seven of their siblings were classified as homozygous for the susceptibility allele (details on genotyping and classification of animals into QTL genotypes given in [22]). DNA from each of these fish was used to construct multiplexed RAD-Seq libraries and sequenced at high depth using a 100 bp paired-end strategy on an Illumina platform. Detailed explanations of the RAD-Seq technique have been given elsewhere [13,14]. However, for an explanation of some of the terminology used in the current study, please refer to the ‘RAD-Seq Terminology’ section of the Methods.

**Figure 1:**
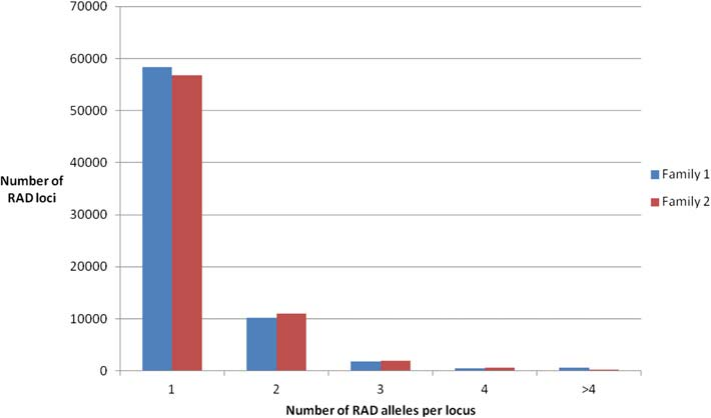
Frequency of single allele and bi-allele and multiple allele RAD loci in the two families.

The experiment was designed to generate very high Illumina sequence coverage for the parents to obtain a clearly defined reference set of RAD alleles to which the lower-coverage offspring sequences could be compared (Table 1). Following read demultiplexing using the sample-specific penta-nucleotide barcodes, there were between 9.2 and 14.0 million reads per parent, and between 1.6 and 6.4 million reads per offspring (Table 1). In total, 123,739 RAD alleles were detected, with 110,303 and 113,639 alleles defined in families 1 and 2 respectively, comprising 100,203 common alleles and 23,536 family-specific alleles. These RAD alleles were clustered into 71,404 and 70,938 RAD loci in families 1 and 2 respectively, of which 69,286 were common (shared at least one allele) (Figure 1, Additional files 1 and 2). As the SbfI restriction site is palindromic, reads are obtained in both directions from the site and, therefore, the number of RAD loci is expected to be twice the number of restriction sites in the genome.

**Table 1:** Experimental design and details of total read counts per individual (after quality control filtering of reads)

**Family 1**						**Family 2**					
**ID**	**IPN phenotype**	**QTL type**	**Library**	**Barcode**	**Total reads (Million)**	**ID**	**IPN phenotype**	**QTL type**	**Library**	**Barcode**	**Total reads (Million)**
Sire	Unknown	RS	1	GAAGC	9.6	Sire	Unknown	RS	4	GGGGA	14.0
Dam	Unknown	RS	1	CTGAA	9.2	Dam	Unknown	RS	4	CGATA	12.4
CR21	Survivor	RR	2	GAAGC	2.9	BR01	Survivor	RR	5	GAAGC	5.7
CR22	Survivor	RR	2	GCATT	1.9	BR02	Survivor	RR	5	GTACA	5.9
CR23	Survivor	RR	2	GTGTG	2.7	BR03	Survivor	RR	5	GTGTG	6.1
CR24	Survivor	RR	2	CTAGG	1.7	BR04	Survivor	RR	5	GCGCC	5.9
CR25	Survivor	RR	3	GGGGA	3.2	BR06	Survivor	RR	6	CTAGG	5.2
CR26	Survivor	RR	3	GTACA	3.3	BR07	Survivor	RR	6	GGAAG	6.4
CR27	Survivor	RR	3	CGATA	3.6	BR08	Survivor	RR	6	CGGCG	5.1
CS31	Mortality	SS	2	GGGGA	2.4	BS11	Mortality	SS	6	CTGAA	5.1
CS32	Mortality	SS	2	GTACA	2.5	BS12	Mortality	SS	6	GTACA	5.6
CS33	Mortality	SS	2	CGATA	2.5	BS13	Mortality	SS	6	GTGTG	5.4
CS34	Mortality	SS	3	CTGAA	3.1	BS14	Mortality	SS	6	GCGCC	5.2
CS35	Mortality	SS	3	GTGTG	2.7	BS16	Mortality	SS	5	GCATT	3.5
CS36	Mortality	SS	3	GCGCC	2.8	BS17	Mortality	SS	5	CTAGG	3.5
CS37	Mortality	SS	3	GGAAG	1.6	BS18	Mortality	SS	5	GGAAG	4.5

### Genome-wide RAD loci

The number of RAD alleles per RAD locus ranged from one (81% of all loci) to 5,764 (Figure 1, Additional files 1 and 2). RAD loci containing many (e.g. hundreds or thousands) RAD alleles are likely to be due to an SbfI site within frequently occurring repeat elements, which are common in the salmon genome [9,31]. A single allele at a RAD locus does not necessarily indicate monomorphism because a polymorphism in the SbfI restriction site itself would still result in an observable presence/absence segregation pattern in the offspring. The second most common type of RAD loci (15%) were those with exactly two RAD alleles (10,238 loci in family 1 and 10,930 loci in family 2), which are likely to contain both SNPs and PSVs. For the current study, we focus on these bi-allelic RAD loci to detect genome-wide SNPs/PSVs, as well as QTL-linked SNPs. A minority of RAD loci (4%) had three or more RAD alleles. These less tractable polymorphisms, possibly the result of polymorphic duplicated regions of the genome (multisite variants), repeat elements containing SbfI sites, multi-allelic SNPs or multiple SNPs, were not considered further in this study. Overall, the frequency pattern of clustering of RAD alleles into RAD loci was similar for the two families (Figure 1).

### Bi-allelic loci containing SNPs and PSVs

To identify candidate bi-allelic segregating SNPs, and to distinguish these from non-segregating bi-allelic PSVs, filtering of RAD loci containing exactly two RAD alleles was performed as described in the Methods. RAD loci where both RAD alleles were present in both parents and all offspring (allowing missing alleles in up to two individuals to account for the possibility of a RAD allele not being sampled in particular individuals due to variation in read coverage) were classified as putative PSVs. We identified 3,768 bi-allelic RAD loci with this excessive heterozygosity in family 1 and 3,491 in family 2, of which 2,674 were common between the two families (Table 2). The overlap between the probable PSV-locus-derived RAD alleles in the two families is expected, since these are likely to reflect fixed differences between homeologous genomic regions. We filtered the remaining RAD loci (4,362 in family 1, 3,646 in family 2, 3,197 in common) to remove those where there was > 3 base divergence between the alleles, as this level of divergence exceeds that expected in simple allelic loci. The final set of high-confidence bi-allelic SNPs (with ≤ 3 base divergence between the pairs of RAD alleles) included 4,725 SNPs identified in 4,111 RAD loci in family 1, and 3,927 SNPs identified in 3,405 RAD loci in family 2. In this final set of bi-allelic RAD markers across the two families there were 6,712 segregating SNPs, of which 1,940 were segregating in both families (Table 2 and Additional file 3). There were an approximately equal number of transitions (cytosine-thymine and adenine-guanine SNPs) and transversions (all other SNPs) in both families (Table 3).

**Table 2:** Details of the bi-allelic RAD loci and filtered putative SNPs and PSVs in Family 1 and Family 2

	**Family 1**	**Family 2**	**Common**^**a**^	**Total**
Total bi-allelic RAD loci	10,238	10,930	6,668	14,500
Bi-allelic RAD loci removed during filtering^b^	2,109	3,804	797	5,116
Remaining bi-allelic RAD loci	8,130	7,127	5,871	9,386
Bi-allelic RAD loci with both alleles fixed	3,768	3,491	2,674	4,585
Segregating bi-allelic RAD loci	4,362	3,636	3,197	4,801
Putative SNPs	4,725	3,927	1,940	6,712

^a^ Common refers to loci where at least one of the RAD alleles at a RAD marker in family 1 matches exactly to one of the RAD alleles at a RAD marker in family 2. In the case of putative SNPs, common refers to the same SNP segregating in both families at the same position in the RAD marker sequence.

^b^ Filtering criteria given in the ‘Methods’.

**Table 3:** Frequency of the six possible nucleotide substitutions at the putative SNPs in Family 1 and Family 2

**SNP substitution (IUPAC ambiguity code)**	**Family 1**	**Family 2**	**Common**	**Total**
C/T (Y)	1,130	932	458	1,604
A/G (R)	1,191	972	498	1,665
G/T (K)	666	552	284	934
A/T (W)	630	531	262	899
A/C (M)	599	538	238	899
C/G (S)	509	402	200	711
Transition	2,321	1,904	956	3,269
Transversion	2,404	2,023	984	3,443
Total	4,725	3,927	1,940	6,712

### Genome-wide segregation patterns

Sire-based linkage clusters of RAD markers were developed by applying a similar approach to Baxter *et al.*[32], and utilising the paucity of male recombination in Atlantic salmon. Within the filtered set of bi-allelic RAD markers, the frequency of segregation patterns of all RAD alleles that were observed in the sire and showed a presence/absence segregation pattern in the offspring (indicating sire heterozygosity) was compared. The most frequently observed segregation patterns are expected to correspond to a set of fully linked RAD markers in these families (i.e. 58 patterns corresponding to the 2n number of chromosomes in Atlantic salmon). For a full explanation of the expected segregation patterns using RADtools refer to ‘RAD Allele Segregation’ in the Methods.

**Figure 2:**
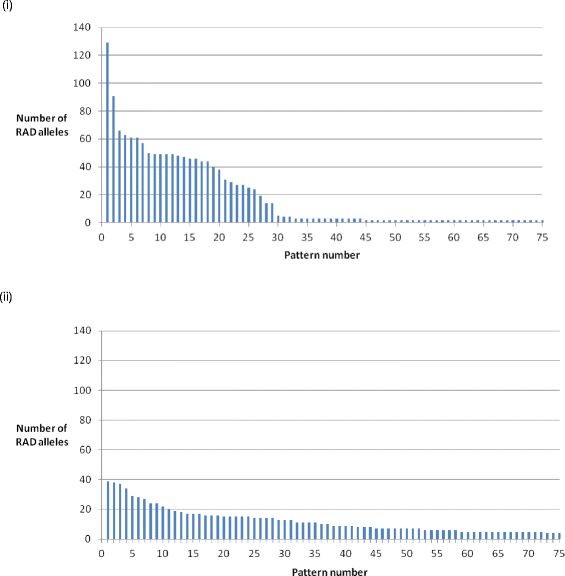
**RAD marker segregation patterns in family 1.** The top 75 most frequently observed RAD marker sire segregation patterns from the filtered bi-allelic loci in family 1. Note the cluster of 29 sire-segregation patterns likely to correspond to RAD markers from regions of distinct linkage groups inherited without recombination in this family. (i) Sire-segregating RAD alleles. (ii) Dam-segregating RAD alleles.

**Figure 3:**
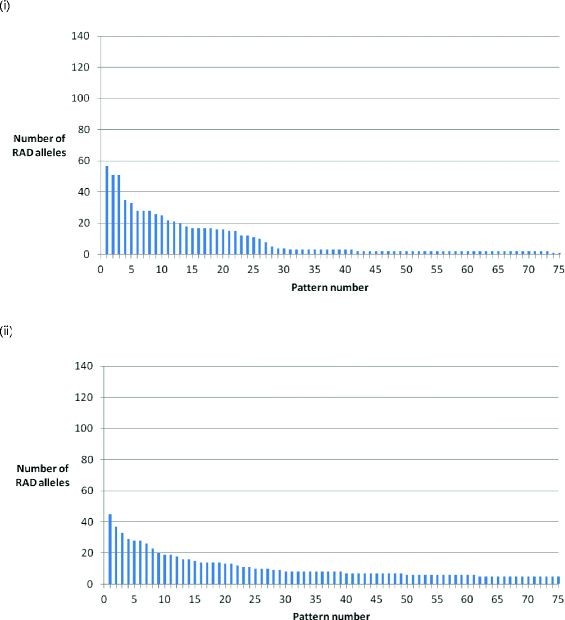
**RAD marker segregation patterns in family 2.** The top 75 most frequently observed RAD marker sire segregation patterns from the filtered bi-allelic loci in family 2. (**i**) Sire-segregating RAD alleles. (**ii**) Dam-segregating RAD alleles.

In family 1, 1,631 sire-segregating RAD markers were identified, of which 1,337 (82%) clustered into the most frequent 58 (29 pairs of) different segregation patterns (Figure 2). The remaining 294 RAD markers displayed 240 different segregation patterns. These data indicate that much of the surveyed genome is inherited without male recombination in this family, although further insight into this phenomenon will require a more detailed SNP genotyping and linkage analysis in larger families. In comparison, the same analysis of dam-based segregation revealed 1,292 segregating RAD markers, of which 602 (47.0%) clustered into the top 29 most frequently occurring pairs of segregation patterns (Figure 2). In family 2, 626 (72%) of the 868 identified sire-segregating markers clustered into the most frequent 27 pairs of segregation patterns (Figure 3), with the remaining 242 markers displaying 202 different segregation patterns. However, due to the overall lower number of segregation patterns in this family, the distinction between patterns likely to correspond to linkage groups and other patterns was not as clear-cut as for family 1 (see Discussion.) Twenty seven pairs of segregation patterns was the most obvious empirical cut-off.

When the RAD marker allele sequences showing the most frequent segregation patterns (linkage clusters) were compared across the two families, matches could be made between specific linkage clusters. Due to the need for sire heterozygosity and dam homozygosity for the segregation pattern at a given polymorphism to be observed (as described under ‘RAD Allele Segregation’ in the Methods), only a proportion of the RAD alleles from any of the linkage clusters would be expected to match between families. For example, even given a minor allele frequency of 0.5, the chance of observing a SNP matching this pattern in any given family is 0.25 [i.e. prob (sire heterozygous) x prob (dam homozygous), or 0.5^2^]. It was possible to match 25 out of the 29 putative RAD linkage clusters in family 1 through this allelic homology to putative linkage clusters in family 2 (Table 4). With only one exception, RAD marker alleles from one linkage cluster in family 1 matched exclusively to one cluster from family 2, and vice versa. This suggests that the linkage clusters are consistent between the two families.

**Table 4:** Details of the sire-based linkage clusters based on the most frequently observed bi-allelic RAD marker allele segregation patterns in the two families

**Putative linkage cluster family 1 (ranked by # of alleles)**	**Corresponding cluster in family 2**^**a**^	**RAD allele count family 1**	**RAD allele count family 2**	**Number of matching RAD alleles between families**
01	02	129	51	20
02	04	91	35	2
03	03	66	51	10
04	08	63	28	12
05	13	61	20	10
06	15	61	17	6
07	05	57	33	8
08	06	50	28	14
09	07	49	28	4
10	11	49	22	12
11	14	49	18	8
12	22	49	15	12
13	12	48	21	8
14	10	47	25	10
15	16	46	17	6
16	27	46	8	1
17	unknown	44	n/a	n/a
18	01	44	57	4
19	unknown	40	n/a	n/a
20	18	38	17	8
21^b^	20	31	16	4
21^b^	26	31	10	2
22	9	29	26	10
23	17	27	17	4
24	21	27	15	2
25	unknown	25	n/a	n/a
26	19	24	16	2
27	24	19	12	6
28^c^	unknown	14	n/a	n/a
29	25	14	11	3

^a^ Cluster ID (number) represents a ranking of segregation patterns in family 2 by number of alleles.

^b^ Two alleles from to linkage cluster 21 in family 1 were found in cluster 26 in family 2.

^c^ IPN QTL-containing chromosome.

### QTL-linked RAD alleles

To identify RAD alleles linked to the IPN resistance locus in the two families, all RAD alleles were screened for segregation patterns that matched the QTL genotype (defined by the microsatellite markers spanning the QTL region) and the IPN mortality phenotype (Table 1). In family 1, there were 90 resistance-linked RAD alleles and 78 susceptibility-linked RAD alleles (Table 5). In family 2, there were 22 resistance and 21 susceptibility-linked RAD alleles. Both resistance and susceptibility-linked RAD alleles were identified in 43 RAD markers in family 1, compared to six RAD markers in family 2, with two of these loci being common across families. For calling QTL-linked SNPs, RAD markers that contain resistance or susceptibility RAD alleles were also screened for RAD alleles that nearly match the QTL genotype pattern (e.g. with two or fewer individuals showing putative recombination.) In total, there were 50 QTL-linked SNPs identified using these criteria, of which four were segregating in both families, and three of these common SNPs were perfectly linked to the QTL. A subset of SNPs with sufficient flanking sequence data for a high-throughput genotyping assay design were identified, and details of all suitable QTL-segregating SNPs in both families and a subset of those only segregating in family 1 (total n = 17) were provided to Kbioscience Ltd (Hoddesdon, Herts) for assay design (Additional file 4). Of these, 13 assays were successful and four failed. From the successful assays, two putative SNPs returned monomorphic genotype data in a test plate of QTL-segregating samples, leaving 11 true segregating RAD SNPs suitable for larger-scale genotyping and linkage analysis.

**Table 5:** RAD alleles and putative SNPs linked to the IPN resistance QTL in families 1 and 2

	**Family 1**	**Family 2**	**Common**	**Total**
Resistance (R) alleles	90	22	10	102
Susceptibility (S) alleles	78	21	6	93
Number of RAD loci containing an R or S allele	114	37	14	137
Number of RAD markers with both R and S alleles	43	6	2	47
Number of putative QTL-linked SNPs	45	9	4	50

### QTL linkage mapping

The 11 IPN QTL-linked SNPs were genotyped across the linkage mapping population (ten full-sib families, total n = 1,341 [22]). Three previously published SNPs [7] and five novel SNPs, closely linked to the QTL (within BAC contig fps378 – see Methods), were also genotyped across the same samples (total = 19 SNPs). 3Sequence details of the SNPs used in the QTL mapping analysis are given in Additional file 4. Finally, an additional two microsatellite markers also from contig fps378 (SSA374 and SSA680) were genotyped across the same samples. These new genotypes were combined with existing data from three microsatellite markers [22] in the mapping analysis. All markers (SNPs and microsatellites) showed highly significant linkage to each other and to the QTL in our mapping population. A dam-based linkage map was then built for the QTL region (Table 6), with markers omitted if the Crimap software could not position the markers in the map. The map covered 37.6 cM which indicates that the QTL-linked markers detected through RAD sequencing were dispersed over a large section of the chromosome.

**Table 6:** Details of the updated dam-based linkage map for the IPN Resistance QTL region on LG 21

**Marker name**	**Marker details/reference**	**Map position (cM)**
RAD_HT09	Additional file 4	0.0
RAD_HT10	Additional file 4	0.8
RAD_HT12	Additional file 4	3.5
RAD_HT02	Additional file 4	3.5
RAD_HT03	Additional file 4	4.9
RAD_HT05	Additional file 4	5.3
RAD_HT04	Additional file 4	6.0
RAD_HT17	Additional file 4	11.1
RAD_HT16	Additional file 4	13.5
Rsa476	Genbank: AY543859	14.3
BHMS217	Genbank: AY544054	16.0
SSA0139ECIG	[7]	17.9
IPN QTL	Grid QTL best estimated position	21.0 (CI 20–22)
RAD_HT01	Additional file 4	22.4
SSA0019ECIG	[7]	22.4
Alu333	Genbank: AY543859	23.6
SSA374	cGRASP linkage map ^a^	24.2
SSA680	cGRASP linkage map ^a^	25.3
fps378_HT03	Additional file 4	26.1
SSA0039ECIG	[7]	26.4
RAD_HT07	Additional file 4	37.6

^a^http://www.asalbase.org/sal-bin/map/index?lg=21&map=Brf-merge.

**Figure 4:**
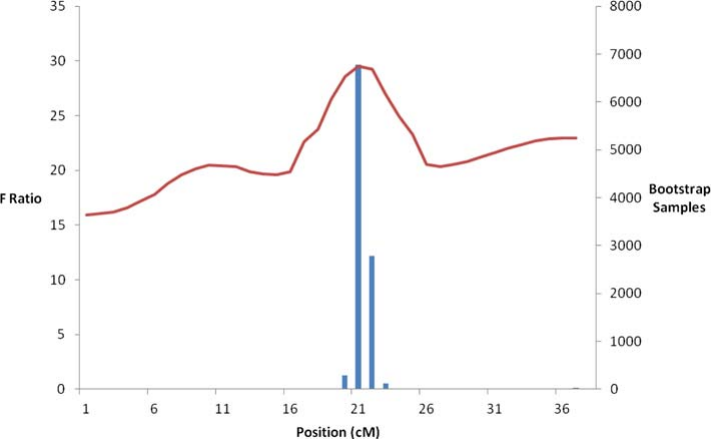
**QTL likelihood profile.** The IPN Resistance QTL likelihood profile on LG 21 with the addition of the new markers. The solid red line is the QTL F Ratio statistic from the linear regression and the blue bars are number of bootstrap samples highlighting the QTL confidence interval.

A dam-based regression interval mapping analysis of the map, genotype and trait (IPN mortality) data was performed using GridQTL [33]. The QTL was mapped to a position of 21 cM on the new map, and the bootstrap analysis (10,000 permutations) gave the average QTL location as 21.3 cM and defined the confidence interval as 2 cM between 20 and 22 cM (Table 6 and Figure 4). The flanking markers for the confidence interval were SSA0139ECIG and RAD_HT01/SSA0019ECIG. These results provide a reduced confidence interval (10 cM down to 2 cM) for the QTL in our populations compared to previous work [15], and a minor reduction compared to study of Moen *et al.* (C.I. 3 cM, [17]).

### SNP-trait association

To assess the population-wide association between the most tightly QTL-linked SNPs (SSA0019ECIG, RAD_HT01, SSA0139ECIG) and IPN mortality, the SNPs were genotyped across a population of 9,000 IPNV-challenged salmon fry from 400 full-sib families deriving from two year groups of the Landcatch Natural Selection broodstock. The markers RAD_HT01 and SSA0139ECIG showed consistent and highly significant association with IPN mortality (and therefore by inference IPN resistance/susceptibility) across the two yearclasses of fish (Table 7), implying linkage disequilibrium between the SNP alleles and the QTL alleles in these populations. SSA0019ECIG showed inconsistent effects in the two year groups (Additional file 5: Table S1) which is likely to be due to its segregation in only a few families (MAF = 0.13.) The contrast in mortality level between the homozygous resistant SNP genotype and the homozygous susceptible SNP genotype was 53% and 46% for RAD_HT01 and SSA0139ECIG respectively.

**Table 7:** **Population-wide association between two closely-linked SNPs in the IPN QTL region and mortality in a freshwater IPNV challenge (all associations significant at P < 0.05)**^**a**^

**2007-strip yeargroup (Ten QTL mapping families)**			
**Mortality Proportion (SE)**			
**SNP**	**RR**	**RS**	**SS**
SSA0139ECIG	0.00 (0.04)	0.22 (0.02)	0.42 (0.02)
RAD_HT01	0.00 (0.03)	0.24 (0.02)	0.54 (0.03)
**2007-strip yeargroup (200 breeding program families)**			
**SNP**	**RR**	**RS**	**SS**
SSA0139ECIG	0.12 (0.02)	0.24 (0.01)	0.47 (0.01)
RAD_HT01	0.11 (0.01)	0.25 (0.01)	0.63 (0.01)
**2006-strip yeargroup (200 breeding program families)**			
**SNP**	**RR**	**RS**	**SS**
SSA0139ECIG	0.10 (0.02)	0.20 (0.01)	0.66 (0.01)
RAD_HT01	0.10 (0.03)	0.17 (0.01)	0.60 (0.01)
**All data combined**			
**SNP**	**RR**	**RS**	**SS**
SSA0139ECIG	0.11 (0.01)	0.24 (0.01)	0.57 (0.01)
RAD_HT01	0.08 (0.01)	0.22 (0.01)	0.61 (0.01)

^a^ For SSA0139ECIG and RAD_HT01 the SNP alleles associated with resistance were thymine and the SNP alleles associated with susceptibility were guanine and adenine respectively.

## Discussion

In this study, RAD-Seq has been used to discover, verify and genotype novel genetic markers in pedigreed Atlantic salmon. By targeting individuals of known disease resistance phenotype and genotype at a major QTL, we discovered and scored novel QTL-linked SNPs with flanking sequence. The use of pedigreed (parent and offspring) samples allowed us to examine segregation of RAD markers, linkage patterns, and to distinguish RAD loci containing putative SNPs from those containing putative paralogous sequence variants (PSVs). The outcomes of the study include a new SNP resource for Atlantic salmon, high-coverage sequence data at sites dispersed throughout the genome, improved knowledge of a genome region harbouring a QTL of major importance to salmon aquaculture and improved population LD-based genetic tests for resistance to IPN.

The RAD library sequence data were analysed with the RADtools pipeline [32]. In this method, unique RAD reads are filtered based on quality score and clustered into RAD loci based on sequence similarity within and across individuals. Further analyses of the data defined putative SNPs and PSVs within RAD loci and examined the segregation patterns of alleles within these loci by looking at presence or absence of alleles in individual animals using methods similar to those used by Baxter *et al.*[32]. These analyses were suitable for our main goals; the thresholds we chose for defining RAD loci and for distinguishing genuine segregation patterns from fluctuations in read counts were empirically derived and conservative. Genotypes in our dataset were defined as ‘presence’ or ‘absence’ of a RAD allele, and as such the RAD markers were effectively acting as dominant markers. Although we did not attempt it in our study, it may be possible to use the fragment count data to differentiate homozygous and heterozygous genotypes, or to identify putative multisite variants based on an excess of one particular allele. Indeed, the recently published software pipeline ‘Stacks’ also detects and genotypes SNPs in short-read sequence data, and uses a maximum likelihood algorithm to call heterozygous and homozygous genotypes based on read counts [34]. This software has recently been used to create linkage maps in the spotted gar [35]. As RAD-Seq continues to develop as a means of genotyping by sequencing, the analysis pipeline is likely to become increasingly robust, standardised and automated, which will broaden its utility and improve consistency.

 A notable outcome from our analyses of the most frequent patterns of segregation was the degree of clustering of sire-based segregation patterns (Figure 2). In one family, 82% of sire segregating RAD markers clustered into the 58 most frequent presence/absence patterns (i.e. two pairs of 29 mirror patterns) which correspond to the number of chromosomes in European Atlantic salmon, without similar clustering in a dam-based analysis. The remaining patterns of segregation may represent male recombination, but are also likely to include patterns that are artefacts due to sequencing errors or false negative allele nulls due to read coverage fluctuation for example. It is well-established that recombination rate is low in regions of the male salmon genome [5-8], and the current data are consistent with an absence of recombination over much of the genome sampled with SbfI in these families. A similar analysis of dam-based linkage patterns in the diamondback moth assigned approximately 65% of RAD markers to 31 pairs of binary patterns, a species with 31 chromosomes and no recombination in females [32]. The most recent salmon linkage map suggests that the differences in recombination between males and females are mainly due to the location of crossovers, which are thought to generally cluster towards the telomeres in males [8]. Therefore, it is likely that the RAD linkage clusters in the current study correspond to non-telomeric regions where male recombination is very low. It is noteworthy that in the recent study of Miller *et al.*[29], the vast majority of identified SNPs in their hybrid rainbow trout populations also clustered towards the putative centromeres. However, the physical distances encompassed by these linkage clusters are unknown, and may include the majority of the chromosome. Our segregation data are based on analyses of two families containing 14 offspring each, and further insight into recombination patterns between the RAD markers will require construction of a linkage map in larger families using the SbfI RAD markers.

 In both the QTL analysis and the bi-allelic segregation pattern analysis, there were notably fewer RAD markers in family 2 compared to family 1. There were some differences in the sequencing technology used for these libraries, and we examined the quality scores and their drop-off by position in the read for both families. Family 1 offspring had marginally better average sequence quality readings than family 2, but the number of RAD alleles defined, the number of RAD alleles per locus, and the number of SNPs were all reasonably consistent between the two families (Figure 1 and Table 2). In the overall unfiltered RAD dataset, there were 6,594 and 5,985 RAD alleles in family 1 that show sire and dam segregation patterns respectively, versus 5,491 and 7,038 in family 2. Therefore, given that sire heterozygosity and dam homozygosity are required to observe a sire-segregation pattern, the differences could reflect greater homozygosity in the family 2 sire and/or greater heterozygosity in the dam. However, there was zero inbreeding for the four parents of these families, making substantial differences in homozygosity unlikely.

Some dissimilarity in RAD marker clustering was also observed between the two families. For example, the top ranked linkage cluster (ranked by number of observed markers) in family 2 was only the 18^th^ largest cluster in family 1, and RAD alleles in linkage cluster 21 in family 1 were split over two linkage clusters in family 2 (Table 4). While these observations may be due to technical bias, it is also possible that they indicate real differences in the rate and/or position of chiasma formation between the two male parents. The extent and pattern of tetravalent pairing in male salmonids and resultant residual tetrasomic manifestations are considered to be influenced by the degree of similarity among the chromosome complement of individuals. Aberrant segregations are thought to be more common in genomes from crosses between genetically divergent individuals [25]. Furthermore, Robertsonian polymorphisms have been observed between and within Atlantic salmon populations with 2n chromosome number thought to vary between 56 and 58 [36]. Therefore, it is possible that genetic heterogeneity, including possible karyotypic differences within the farm strain could explain some of the differences between the families.

 A subset of QTL-linked SNP markers were genotyped at a population level and assessed for linkage and association with IPN mortality. Previous studies by our group [15] and Moen *et al.*[17] have mapped the IPN-resistance QTL to a region of linkage group 21 with a confidence interval of 10 cM and 3 cM respectively. In the current study, the genotyped QTL-linked RAD SNPs were spread across a large region of our linkage map (37.6 cM) and the QTL confidence interval was narrowed marginally to 2 cM. In the study of Moen *et al.*[17], microsatellite marker haplotypes showing population-level association with IPN mortality were identified by establishing the phase-relationship between the QTL allele and the marker haplotype in QTL-heterozygous parent [17]. However, several different marker haplotypes were associated with a particular QTL allele which hinders the practical application of population LD- based selection. Here we demonstrate that a RAD-derived SNP (RAD_HT_01) and a previously published SNP (SSA0139ECIG [7]) show highly significant population-level association with IPN mortality, implying strong LD between these SNPs and the QTL in the Landcatch Natural Selection broodstock population. We do not know how physically close these SNPs are to the QTL causal mutation, and the level of LD is likely to vary from population to population. The short timescale and cost-efficiency of our RAD-Seq approach highlights its utility for QTL-linked marker generation and fine-mapping. Additional QTL-linked RAD markers can be generated by using a different restriction enzyme, and the RAD approach we applied herein can be applied to map loci affecting other economically important traits.

## Conclusions

We have used RAD-Seq in pedigreed Atlantic salmon to discovery, verify and genotype novel markers dispersed throughout the genome, including SNPs linked to a major QTL. These markers are likely to be important for future salmonid genomics research, and will have applications in aquaculture for selective breeding. Integration with existing salmon genome maps is a prerequisite for this, and is underway. We have discovered 50 segregating SNP markers linked in at least one family to a major QTL affecting resistance to the viral disease IPN, and have used high-throughput genotyping assay for a subset of markers to identify those with population-wide utility as tests for resistance to the disease. RAD-Seq of pooled animals of disparate phenotypes or QTL genotypes is likely to have broad utility for mapping the genomic regulation of important quantitative traits in a cost and time efficient manner.

## Methods

### Animals and disease challenge experiment

The fish used in the RAD sequencing experiment were a subset of the population described elsewhere [22]. Briefly, twenty families from the breeding nucleus of Landcatch Natural Selection Ltd at Ormsary, Scotland were transported to the Centre of Environment, Fisheries and Aquaculture Science (Cefas) at Weymouth, England as fertilised eggs. Two replicate tanks of ~100 fry per family were bath-challenged with virulent IPNV, alongside a mock-challenged tank of ~100 fry per family. Mortalities due to IPN were recovered, and the experiments were terminated once mortalities were negligible and survivors sampled. The ten families with the highest IPN mortality level were then used for QTL mapping using microsatellite markers. In the current study, families 1 and 2 correspond to families C and B in [22], and were established as having both parents heterozygous for the QTL. All four parents, plus seven QTL homozygous resistant, and seven QTL homozygous susceptible offspring were chosen from each family for the RAD sequencing experiment. The sex of the offspring was unknown. The experiments were performed with a UK Home Office license and under approval of the Cefas ethical review committee.

### RAD-seq terminology

The purpose of this section is to define some of the terms used in the current study. A **read** is an individual raw sequence of a fragment of DNA; reads can be single or, in our study, paired-end (reads determined from both ends of the fragment). Reads were processed using the RADtools software pipeline [32]. Reads with identical sequences at both ends are collapsed into **fragments** to remove PCR duplicates, corresponding to unique DNA fragments in the initial sheared genomic DNA sample. A **RAD locus** is a collection of one or more fragments, theoretically corresponding to all fragments from the genomic region either upstream or downstream of a particular SbfI restriction site. Each RAD locus contains one or more **RAD alleles**, depending on whether or not the locus contains polymorphic/paralogous variation within and/or across individuals. Each RAD allele has a read count and a fragment count in each individual. A **RAD marker** is a polymorphic RAD locus segregating in the analysed population(s). A **segregation pattern** is a binary pattern of presence/absence of a particular RAD allele at a RAD marker across all sampled individuals. A **linkage cluster** is a group of RAD markers with a common segregation pattern.

### RAD library preparation and sequencing

DNA was extracted from caudal fin tissue using the REALPure genomic DNA extraction kit (Durviz S.L.) and treated with RNAse to remove residual RNA. Each sample was quantified by spectrophotometry (Nanodrop) and its quality assessed by agarose gel electrophoresis, and was then diluted to a concentration of 50 ng/μL in 5 mM Tris, pH 8.5. The RAD library preparation protocol followed closely the methodology originally described in [13] and latterly comprehensively detailed [37]. The RAD specific P1 & P2 paired–end adapters and library amplification PCR primer sequences used in this study are detailed in [28]. Briefly, each sample (1.5 μg parental DNA/0.5 μg offspring DNA) was digested at 37°C for 30 min with SbfI high fidelity restriction enzyme (New England Biolabs -NEB) using 6U SbfI per μg genomic DNA in 1× Reaction Buffer 4 (NEB) at a final concentration of 1 μg DNA per 50 μL reaction volume. The reactions (75 μL/25 μL final volumes for parental/offspring samples respectively) were then heat inactivated at 65°C for 20 minutes. Individual specific P1 adapters, each with a unique 5 base barcode (Table 1), were ligated to the SbfI digested DNA at 22°C for 30 minutes by adding 3.75/1.25 μL 100 nM P1 adapter, 0.9/0.3 μL 100 mM rATP (Promega), 1.5/0.5 μL 10× Reaction Buffer 2 (NEB), 0.75/0.25 μL T4 ligase (NEB, 2 M U/mL) and reaction volumes made up to 90/30 μL with nuclease free water for each parental/offspring sample. Following heat inactivation at 65°C for 20 minutes, the ligation reactions were then combined in appropriate multiplex pools (either two parental samples or seven offspring samples per library pool; Table 1). For each library pool 100 μL (c. 2 μg digested DNA) was sheared to c. 150–700 bp size range (Covaris sonicator). The sheared DNA was column purified (PCR MinElute Kit, Qiagen), being eluted in 35 μL EB buffer (Qiagen). Each of the six library samples were then size selected (c. 250–500 bp) by gel electrophoresis (0.5× TAE; 1.1% gel). Gels were run (2 V/cm for 10 min; 8 V/cm for 50 min) in ice-cold buffer – to minimise small fragment diffusion. Marker lanes (100 bp ladder) were cut out of the gel by scalpel, quickly stained with EtBr, viewed under UV, and the appropriate size range flagged by ‘nicking’ the marker lane. The gel was then reassembled and the identified size selected band was excised using a clean scalpel blade. In this way, the size-selected DNA was not exposed to EtBr or UV radiation.

The remainder of the library construction (i.e. gel purification; end repair, dA overhang addition, P2 paired-end adapter ligation and library amplification) followed the original protocol [13,37] exactly. A total of 150 μL of each amplified library (16–18 PCR cycles) was prepared, column purified, eluted in 35 μL EB buffer and size selected (c. 300–550 bp) by gel electrophoresis, as described above. Following a final gel elution step into 20 μL EB buffer (MinElute Gel Purification Kit, Qiagen), the libraries were QCed by electrophoresis (Bioanalyser, Agilent) and accurately quantified by fluorimetry. One parent library and two offspring libraries were produced from each family. Each library was sequenced (100 base pair paired-end reads) on the Illumina GAIIx or HiSeq 2000 platform at the GenePool Genomics Facility, University of Edinburgh (http://genepool.bio.ed.ac.uk).

### Generating candidate RAD loci and RAD alleles

Raw Illumina reads were processed into candidate RAD loci using the open source RADtools pipeline (http://www.radseq.info) and following the protocol described in Baxter *et al.*[32]. Briefly, loci were inferred for each family separately as follows: reads were separated by barcode using the programme ‘*RADpools*’, candidate RAD loci were inferred for each individual in a family using ‘*RADtags*’ with a cluster distance of nine (allowing for up to nine base mismatch between reads). Loci were then merged across all individuals within each family with ‘*RADmarkers*’, merging loci with shared RAD alleles, allowing up to three mismatches between alleles. PCR duplicates were removed by collapsing reads with identical sequences at both ends into a single unique sequence (a fragment). We ran analyses with a minimum threshold of fragment coverage per allele (discarding RAD alleles with fewer than five fragments), but the results presented herein are from analyses of all RAD alleles for which at least one fragment was observed.

### SNP discovery

The full set of RAD loci was filtered to include only those with exactly two RAD alleles in each family. These bi-allelic loci were excluded from further analysis if they were considered likely to contain errors as indicated by (i) either allele was absent in both parents, (ii) fewer than two (of 14) offspring inherited an allele at the locus, (iii) the average fragment count for an allele within the locus was lower than ten in the parents [average parental fragment count was 38.7 (family 1) and 31.2 (family 2)], (iv) the average fragment count for an allele within the locus was lower than five in the offspring [average offspring fragment count was 13.3 (family 1) and 11.2 (family 2)], (v) any animal contained neither allele. Bi-allelic RAD loci from this subset were identified as containing putative PSVs if both RAD alleles were present in all 16 individuals). RAD loci where both alleles were present in all 16 individuals, except for one or two absences, were included in this list as it is very unlikely that these are segregating loci, and very likely that particular individuals were not sequenced for an allele by chance due to fluctuating read counts across individuals and loci. After removal of putative PSVs, the two alleles within each remaining RAD locus were aligned and the number of differences between them counted. Bi-allelic RAD loci with between one and three mismatches (putative SNPs) were then converted to single sequences with IUPAC ambiguity codes as the final set of filtered non-repeat region SNPs.

### RAD allele segregation

Within the filtered bi-allelic dataset, each RAD allele was scored as present (1) or absent (0) in all sequenced animals of a full-sibling family, thus producing a 16-digit binary string per allele (corresponding to the two parents and two groups of seven offspring in each family) which is the segregation pattern. At each bi-allelic sire-heterozygous RAD marker, the grandpaternal sire allele will be transmitted to one group of offspring, and the grandmaternal sire allele will be transmitted to the remaining offspring, resulting in two ‘mirror’ segregation patterns per marker (see [32]). However, only a subset of linked RAD markers will result in observable segregation patterns and, of this subset, only one of the two mirror patterns will be seen. Observing a sire-segregation pattern is dependent on the dam being fixed for one of the alleles at the RAD marker because there is no means to differentiate between homozygous and heterozygous offspring using this method. In the remaining linked RAD markers, to explain why only one of the two ‘mirror’ segregation patterns is observed, consider RAD markers containing a single bi-allelic SNP where the sire is heterozygous AB, and ‘A’ is the grandpaternal sire allele and ‘B’ is the grandmaternal sire allele. If the dam is homozygous BB, only the A (grandpaternal) sire allele will show the visible segregation pattern. For another sire-heterozygous RAD marker containing a single SNP on the same chromosome, the dam may be homozygous AA, in which case only the B (grandmaternal) sire allele will show the visible segregation pattern. Therefore, a subset of fully-linked sire-heterozygous and dam-homozygous RAD markers will fall into one of two ‘mirror’ sire segregation patterns depending on whether the visible allele is on the grandpaternal or grandmaternal sire chromosome, which in turn depends on which allele is fixed in the dam. For comparison, the analysis was repeated for alleles present in the dam but not the sire. The text and sequence file analyses were performed using custom Perl scripts (Additional files 6 and 7) and the online genomics resource ‘Galaxy’ [38].

### BAC-end sequencing

The BAC contig fps378 was identified as being closest to the IPN QTL region based on the aligned linkage and physical maps given in (http://grasp.mbb.sfu.ca/[39]). Using BAC-end sequence data, PCR primer sets were designed using Primer3 [40] to amplify short regions (predicted to be from 405 to 686 bp) dispersed along the BAC contig (Additional file 8: Table S2). These regions were PCR amplified in the four parental samples from family 1 and 2, and sequenced on an ABI 3730xl instrument at the ARKGenomics laboratory at the Roslin Institute (Edinburgh, UK; http://www.ark-genomics.org/.) SNPs were identified by aligning the sequences using ClustalW [41].

### Linkage and QTL analysis

QTL-linked SNPs were defined from a list of RAD loci containing allele segregation patterns matching the QTL genotype (allowing up to two discordances). Details of a subset of 17 SNPs with sufficient flanking sequence were sent to Kbiosciences (Hoddesdon, Herts) for high-throughput SNP assay design, and the QTL mapping population [22] were genotyped for all SNPs. Paired-end contigs (see [42]) were developed for the RAD loci containing these SNPs to provide additional flanking sequence information. Microsatellite markers SSA374 and SSA680 (also from BAC contig fps378 and therefore close to the QTL) were genotyped in a multiplex PCR across the population after optimising fragment amplification on a TProfessional Gradient thermocycler (Biometra, Gottingen, Germany) and using an ABI-377 mediated fluorescent detection to create allelic profiles. A linkage map of the QTL region was constructed using Crimap Version 2.4 [43]. A ‘twopoint’ analysis was initially used to calculate the pairwise linkage between markers. Due to a lack of male recombination, only informative dam meioses were used for determining marker order and position using a ‘build’ analysis. A ‘flipsn’ analysis was then used to verify that the obtained marker order was the most likely order. The GridQTL software was then used to calculate the most likely QTL position. The significance threshold was calculated empirically by permutation analysis (10,000 permutations), and the confidence interval for the QTL was defined using a bootstrapping approach (10,000 bootstrap samples.)

### SNP-trait association and population-wide verification

Two separate IPNV challenge trials of families from the breeding nucleus of Landcatch Natural Selection Ltd (LNS) were performed at Cefas, Weymouth, UK. The two groups of families were from the yeargroups of the LNS broodstock stripped in 2006 and 2007, disease challenged as fry (approximately two months post-hatching) in 2007 and 2008 respectively. Details on the challenge protocol are given in [23,44]. Briefly, for each yeargroup, there were two mixed-family challenge tanks. The tanks comprising fry from all families were given an immersion IPNV challenge with V0512-1 serotype Sp A2. The challenge experiment was run until IPN-related mortalities had ceased which was 42 days post-challenge. Overall mortality rates due to IPN were 41% in the 2006-strip yeargroup (total n = 4,846) and 29% in the 2007-strip yeargroup (total n = 5,247). Samples were obtained from mortalities and survivors and fry were assigned to family using an in-house microsatellite multiplex genotyping panel. The most tightly linked SNPs to the QTL on LG 21 were successfully genotyped in 9000 animals. The association between SNP genotype and the binary trait of IPN mortality was assessed using a REML model with tank and family fitted as fixed effects in Genstat [45].

## Competing interests

The authors declare they have no competing interests.

## Authors’ contributions

RDH, JWD, SCB, KG, MLB, JEB and JBT conceptualised and designed the RAD sequencing experiment. JWD and RDH analysed the data. RDH wrote the manuscript. NRL sequenced and discovered BAC end SNPs. MLT contributed to the RAD library preparation and sequencing. KG planned and coordinated the sequencing of RAD libraries. JBT prepared the RAD libraries. JCM and AH performed the microsatellite genotyping. JCM, AH, DRG and AET designed and managed the population-wide association and verification experiment and its associated genotyping. All authors read and approved the final manuscript.

## Accession numbers

Raw RAD sequence data from this article have been deposited in the European Nucleotide Archive under accession number ERP001162.

## Supplementary Material

Additional file 1 Details of Family 1 RAD loci, number of RAD alleles and fragment counts for all individuals. Click here for file

Additional file 2 Details of Family 2 RAD loci, number of RAD alleles and fragment counts for all individuals. Click here for file

Additional file 3 List of RAD allele sequences containing SNPs marked using IUPAC ambiguity code. Click here for file

Additional file 4 List of high-throughput assay SNPs used for QTL mapping and population-wide association analysis. Click here for file

Additional file 5 **Table S1.** Details of the primers and SNPs for the BAC contig fps378. Click here for file

Additional file 6 **Perl script to identify the most frequent segregation patterns in the RAD loci (Additional files ****1****and****2****).** Click here for file

Additional file 7 **Perl script to merge SNP-containing bi-allelic RAD loci into a single list of sequences with IUPAC ambiguity code (Additional file ****3****).** Click here for file

Additional file 8 **Table S2.** Population-wide association between genotype at SSA0019ECIG and mortality in a freshwater IPNV challenge (all associations significant at P<0.05). Click here for file
