# Supplementary material for: Characterisation of QTL-linked and genome-wide restriction site-associated DNA (RAD) markers in farmed Atlantic salmon
Source: BMC Genomics. 2012 Jun 15;13:244. doi: 10.1186/1471-2164-13-244 (PMC3520118; doi:10.1186/1471-2164-13-244)
Supplement: Additional file 5 — Table S1. Details of the primers and SNPs for the BAC contig fps378. [file 1471-2164-13-244-S5.doc]

**Additional file5: Population-wide association between genotype at SSA0019ECIG and mortality in a freshwater IPNV challenge (all associations significant at P<0.05)**

|  | **Mortality Proportion (SE)** | | |
| --- | --- | --- | --- |
|  |  |  |  |
| 2007-strip yeargroup (Ten QTL families) | **TT** | **TG** | **GG** |
|  | 0.24 (0.01) | 0.49 (0.04) | N/A |
| 2007-strip yeargroup (200 families) | **TT** | **TG** | **GG** |
|  | 0.29 (0.01) | 0.25 (0.03) | 0.72 (0.15) |
| 2006-strip yeargroup (200 families) | **GG** | **TG** | **TT** |
|  | 0.02 (0.07) | 0.11 (0.02) | 0.47 (0.01) |
| All data combined | **TT** | **TG** | **GG** |
|  | 0.37 (0.005) | 0.20 (0.02) | 0.16 (0.06) |
|  |  |  |  |
