# Supplementary material for: Characterisation of QTL-linked and genome-wide restriction site-associated DNA (RAD) markers in farmed Atlantic salmon
Source: BMC Genomics. 2012 Jun 15;13:244. doi: 10.1186/1471-2164-13-244 (PMC3520118; doi:10.1186/1471-2164-13-244)
Supplement: Additional file 8 — Table S2. Population-wide association between genotype at SSA0019ECIG and mortality in a freshwater IPNV challenge (all associations significant at P<0.05). [file 1471-2164-13-244-S8.doc]

Additional file 8: Details of the primers and SNPs for the BAC contig fps378

| **SNP ID** | **BAC End Sequence ID** | **Forward Primer** | **Reverse Primer** | **Amplicon Size (bases)** | **SNP** | **Position in Sequence** |
| --- | --- | --- | --- | --- | --- | --- |
|  |  |  |  |  |  |  |
| fps378_HT_01 | S0032J24 3' | CTTCACAGTCCCCACTCTGG | GGTTGCCTTGATTCCTCATC | 625 | C/T | 514 |
| fps378_HT_02 | S0001J08 5' | TACTCCATCAGCCGCCTAAC | CAAAGCCCCAAAAATTTGAA | 433 | A/T | 85 |
| fps378_HT_03 | S0053A14 5' | AGCTGACACCCCAGCTCTTA | TGAAGTTCTGGCAATTTCAATG | 501 | G/T | 145 |
| fps378_HT_04 | S0124D16 5' | CTATTACCCAACCCGTCCAA | GGCACAATGAGCCAACATAA | 628 | A/T | 70 |
| fps378_HT_05 | S0124D16 5' | CTATTACCCAACCCGTCCAA | GGCACAATGAGCCAACATAA | 628 | A/T | 180 |
|  |  |  |  |  |  |  |
